# Prefrontal and anterior cingulate cortex abnormalities in Tourette Syndrome: evidence from voxel-based morphometry and magnetization transfer imaging

**DOI:** 10.1186/1471-2202-10-47

**Published:** 2009-05-12

**Authors:** Kirsten R Müller-Vahl, Jörn Kaufmann, Julian Grosskreutz, Reinhard Dengler, Hinderk M Emrich, Thomas Peschel

**Affiliations:** 1 Clinic of Psychiatry, Socialpsychiatry and Psychotherapy, Hannover Medical School, Hannover, Germany; 2 Department of Neurology, Hannover Medical School, Hannover, Germany; 3 Department of Neurology II, Center for Advanced Imaging (CAI), Otto-von-Guericke-University Magdeburg, Magdeburg, Germany; 4 Department of Neurology, University of Jena, Jena, Germany; 5 Center for Systems Neurosciences (ZSN), Hannover, Germany

## Abstract

**Background:**

Pathophysiological evidence suggests an involvement of fronto-striatal circuits in Tourette syndrome (TS). To identify TS related abnormalities in gray and white matter we used optimized voxel-based morphometry (VBM) and magnetization transfer imaging (MTI) which are more sensitive to tissue alterations than conventional MRI and provide a quantitative measure of macrostructural integrity.

**Methods:**

Volumetric high-resolution anatomical T1-weighted MRI and MTI were acquired in 19 adult, unmedicated male TS patients without co-morbidities and 20 age- and sex-matched controls on a 1.5 Tesla neuro-optimized GE scanner. Images were pre-processed and analyzed using an optimized version of VBM in SPM2.

**Results:**

Using VBM, TS patients showed significant decreases in gray matter volumes in prefrontal areas, the anterior cingulate gyrus, sensorimotor areas, left caudate nucleus and left postcentral gyrus. Decreases in white matter volumes were detected in the right inferior frontal gyrus, the left superior frontal gyrus and the anterior corpus callosum. Increases were found in the left middle frontal gyrus and left sensorimotor areas. In MTI, white matter reductions were seen in the right medial frontal gyrus, the inferior frontal gyrus bilaterally and the right cingulate gyrus. Tic severity was negatively correlated with orbitofrontal structures, the right cingulate gyrus and parts of the parietal-temporal-occipital association cortex bilaterally.

**Conclusion:**

Our MRI *in vivo *neuropathological findings using two sensitive and unbiased techniques support the hypothesis that alterations in frontostriatal circuitries underlie TS pathology. We suggest that anomalous frontal lobe association and projection fiber bundles cause disinhibition of the cingulate gyrus and abnormal basal ganglia function.

## Background

Tourette syndrome (TS) is defined as a childhood-onset disorder characterized by motor and vocal tics often associated with behavioural problems. Findings from in vivo neuroimaging studies provided evidence that cortico-striatal-thalamo-cortical circuits are pathophysiologically involved. Most recent *volumetric *MRI studies in TS investigated basal ganglia size and demonstrated reduced [1,2] or normal [3-5] volumes with [1,3,4] or without [2,5] loss of normal basal ganglia asymmetry. There is evidence that basal ganglia volumes are influenced by numerous factors such as age [2], TS symptoms and severity [1,6,7], gender [5], co-morbid attention-deficit hyperactivity disorder (ADHD) [4,8], streptococcal infections [9,10], and neuroleptic medication [2]. Using volumetric MRI to investigate frontal regional brain volumes, a significant age effect with larger prefrontal volumes in children, but smaller volumes in adults has been detected [11]. When gray and white matter compartments were segmented, in boys-only groups increases in right frontal white matter volumes [12], and decreases in the left deep frontal white matter were found [13]. Using automated voxel-based morphometry (VBM) [14], in contrast, in adult TS patients increased gray matter volumes in the left midbrain were found, while in TS boys increases in gray matter volumes were described in the ventral putamen bilaterally, but decreases in the left hippocampal gyrus [15]. Since the same whole-brain-based technique of optimized VBM had been used, discrepancies might be related to effects of age, gender, co-morbidities, and drug exposure [16].

The aim of the present study was to investigate regional gray and white matter brain abnormalities in TS compared to normal controls using VBM, an objective unbiased whole-brain approach to characterize differences in gray and white matter volumes, and magnetization transfer imaging (MTI) which may be more sensitive than conventional volumetric imaging to subtle structural brain changes in TS.

MTI is a nuclear magnetic resonance technique relying on the transfer of energy between highly bound protons within structures as myelin and the very mobile protons of free water. In brain, the major macromolecules in the bound proton pool are the cell membrane proteins and phospholipids in gray matter and myelin in white matter. Bound protons which are undetected by conventional MRI because of their very short relaxation times, can be preferentially saturated using an off-resonance radio frequency pulse. This leads to a reduction in signal intensity, which is dependent on macromolecular density. These changes can be quantified by the magnetization transfer ratio (MTR). MTR correlates with in vivo measurements of N-acetyl-aspartate, a marker of neuronal integrity [17], and MTR reductions correlate with myelin and axonal loss in the white matter in post-mortem tissue, and *in vivo *in a range of neurological diseases [18]. In gray matter, Wallerian degeneration triggered by distant axonal damage and microscopic lesions is thought to explain cortical MTR reductions in multiple sclerosis [19]. So far, this imaging technique has not been used in TS patients. Both, VBM and MTI are analyzed on a voxel-by-voxel basis with no need for manually placed regions of interest (ROI) and an *a priori *hypothesis.

To exclude influences from age, gender, medication, and behavioural disorders, we investigated 19 adult, male, unmedicated TS patients without co-morbidities ("TS only") and 20 age- and sex-matched normal controls. Based on recent data obtained from both volumetric and functional [16,20] neuroimaging studies, we hypothesized that frontal cortices, limbic structures, the striatum, parts of the corpus callosum and parietal-temporal-occipital association cortices may be altered in adult TS patients.

## Methods

### Patients

19 unmedicated male TS patients (mean age = 30.4, range 18–60, according to DSM-IV-TR criteria) and 20 age- and sex-matched control subjects (mean age = 31.7, range 18–65, p = 0.71) were enrolled in the study. All patients were investigated by one of the authors (KR MV), who is experienced in the diagnosis of TS. Twelve patients were drug-naïve, 6 patients were drug-free for at least 1 year (one for 5 months) before entering the study. Using the Yale Global Tic Severity Scale (YGTSS) [21] mean disease severity was 28.8 (range, 9–69). For diagnosing additional obsessive-compulsive disorder (OCD) and ADHD we used both a clinical interview and the German version of the Yale-Brown obsessive compulsive scale (Y-BOCS) [22] and the short form of the German version of the Wender Utah rating scale (WURS-k) [23], respectively. Using the Y-BOCS, we found no or subclinical OCD in 18 patients and mild OCD in one patient. When using the WURS-k, results of 4 patients suggested the diagnosis of ADHD. However, none of these patients fulfilled diagnostic criteria for ADHD (according to DSM IV-TR), neither at present time nor in childhood. None of the patients suffered from other co-morbid disorders such as depression, anxiety, addiction, or self-injurious behaviour. Physical and neurological examination and routine blood laboratory tests were normal. This study was approved by the local ethic committees of the Hannover Medical School and the Otto-von-Guericke-University Magdeburg. After complete description of the study to the subjects, written informed consent was obtained.

### Data acquisition

Images were acquired on a neuro-optimized 1.5-T GE Signa Horizon LX (General Electric Company, Milwaukee, WI, USA) using a 3-dimensional T1-weighted spoiled gradient recalled echo (SPGR) sequence generating 124 contiguous sagittal slices (RT 24 ms; TE 8 ms; flip angle 30°, 2 averages, acquisition time 13'10", in plane resolution 0.97 × 0.97 × 1.5 mm^3^). The protocol for MTI consisted of a proton density (PD)-weighted SE sequence (TR 2600, TE 20, 256 × 256) both with (MT) and without (non-MT) a preparing saturation pulse (1200 Hz off-resonance, 1180° flip-angle, 16 ms). Forty-eight slices of 3 mm thickness aligned along the AC-PC line were acquired. Image post-processing included a simple intersequence correction of movement with the automated image registration package based on rigid body model (AIR) [24] and calculation of MTR maps pixel-by-pixel according to the following formula: MTR = ([non-MT - MT]/non-MT) × 100.

During scanning, all participants were comfortably placed and their heads were fixated within the headcoil with special cushions. All subjects received additional T2-weighted images.

### Pre-processing of structural data

Data were processed on a standard IBM-compatible PC using SPM2 statistical parametric mapping software (Welcome Department of Cognitive Neurology, London) and working in an analysis environment (MATLAB; the Math Works Inc, Natick, Mass). The images were reoriented into oblique axial slices aligned parallel to the anterior-posterior commissural axis with the origin set to the anterior commissure.

### Data pre-processing for voxel-based morphometry

An optimized version of the VBM protocol was followed as recently described by our group [25,26]. The resulting images were resliced to a final voxel size of 1 mm^3^. Voxel values in segmented images of gray and white matter were multiplied by the Jacobian determinants derived from spatial normalization to provide intensity correction for induced regional volumetric changes, thus preserving within-voxel volumes that may have been altered during non-linear normalization. These 'modulated' images were smoothed to 8 mm using a FWHM (full width half-maximum) Gaussian filter to minimize individual gyral variations and to increase the statistical validity of the analysis.

### Data pre-processing for voxel-based MTI

The main challenge facing voxel-based MTI analysis involves meeting the requirement for an optimal matching of the brains being compared. Therefore, a complex pre-processing procedure was employed as described before [27] consisting of the creation of a series of templates in order to derive the best possible parameter set for normalization. To achieve maximum precision, a proton density (PD)-weighted template with scanner specific image contrast best adapted for the studied sample population was created. Therefore, all PD-weighted scans were normalized to the PD-template provided by SPM2 and subsequently smoothed with an 8-mm isotropic FWHM isotropic Gaussian kernel. Thus, a mean image was created that served in the following as sample population and scanner specific template and all PD-images in native space were normalized to it.

To minimize the influence of other structures than the brain on the registration, the normalized images were skull-stripped using the segmentation procedures implemented in SPM2 that included the brain extraction step. The resultant white and gray matter partitions were summed and thresholded at 0.15 to create a binary mask image that contained 'ones' for all gray or white matter voxels with a value greater than 0.15 and 'zeros' for all voxels belonging to non-brain tissue. This mask image was then multiplied voxel-by-voxel with the corresponding normalized PD-images, thus discarding the majority of extracerebral tissue and CSF while preserving the original voxel intensities. The obtained normalized skull-stripped images were then smoothed with an 8 mm isotropic FWHM Gaussian kernel and a new skull-stripped PD-weighted template was created.

The PD-images in native space (non-normalized) were subject to exactly the same procedure, in which they were skull-stripped, segmented, multiplied with a binary mask and then normalized to the previously skull-stripped PD-weighted template. This prevented any contribution of non-brain voxels and afforded optimal spatial normalization of the individual PD images and provided an optimized normalization parameters set for PD images adapted to the investigated population sample. As the segmentation of native images is performed on affine-normalized images, but the probability maps used as Bayesian priors for segmentation are in stereotactic space, the optimized normalization parameter set were reapplied to the original PD weighted images in native space. These optimally normalized PD-images – now in stereotactic space – were then again skull-stripped by using the procedure described above. This resulted in optimally normalized PD-images removed from extracerebral tissue and CSF.

Finally, the optimized normalization parameter set was applied to the inherently co-registered MTR-maps in native space and resliced with a final voxel size of 1 mm^3^. The normalized MTR-images were then skull-stripped applying the corresponding brain mask derived from the optimally normalized PD-weighted images. In analogy to the PD-images, this resulted in optimally normalized MTR-maps removed from non-brain structures. Images were smoothed to 8 mm using a FWHM Gaussian filter to improve signal-to-noise ratio.

### Statistical analysis

Processed images of each tissue class were analyzed in the framework of the general linear model. For VBM, group comparison of TS patients and healthy controls was performed in SPM2 using the model 'compare-populations: one scan/subject (ANCOVA)'. During modulation we incorporated the correction for volume change induced by spatial normalization. Therefore, it was appropriate to include the global mean voxel value of each tissue class as a covariate to determine the regionally specific pattern of loss or gain within each compartment as well as to remove any variance due to differences in head size. Additionally, regression analyses with clinical measures were explored for the TS patients using the SPM2 model 'single subjects: covariates only'. As for the group comparisons, ANCOVA with the mean voxel value was used to normalize image intensity in the different tissue maps to allow identification of the regional pattern of these correlations.

MTR maps were analyzed separately using the model 'compare-populations: one scan/subject (two sample t-test)'. Only voxels exceeding an absolute threshold of 15% were included in the analysis to minimize low signal-to-noise. Regression analyses in the patients were explored using the model 'simple regression (correlation)'.

Resulting statistical parametric maps of VBM and MTR were derived at a significance level of p < 0.001, uncorrected with an extent threshold of 10 voxels.

For regions where an effect was hypothesized, namely the fronto-striatal and limbic system (see introduction), a small volume correction (SVC) limited to the volume of that particular region was performed using a sphere of 15 mm radius [28]. Here, we controlled for multiple comparisons by using the family wise error (FWE) method (p < 0.05).

## Results

On visual inspection of the MR images no subject had focal atrophy of any brain region or movement artefacts which may have hindered alignment into standard space or segmentation into gray and white matter. Furthermore, there were no overt involuntary movements, which were monitored during scanning by one of the investigators (TP). Mean (± SD) intracranial volume did not significantly differ between patients with TS and control subjects (1.31 l ± 0.12 and 1.35 l ± 0.12; p = 0.16).

### Group comparisons

#### Voxel based morphometry

**Figure 1:**
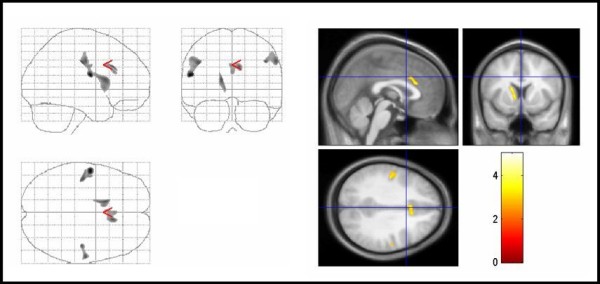
**Areas of decreased regional gray matter volumes in TS patients compared with controls**. Areas of decreased regional gray matter volumes in TS patients compared with controls (thresholded at uncorrected P < .005 for display purpose only). The three orthogonal planes on the left side represent a typical maximum intensity projection "glass brain", and the set of images on the right side illustrates results superimposed on averaged and normalized T1-weighted images of the study population in selected planes. The images are shown in neurological convention. The color bar represents the *t*-score. Significant voxels were found in the left caudate nucleus, cingulate gyrus, primary and premotor cortex bilaterally (corrected P < .05). TS = Tourette syndrome.

Compared with healthy controls, TS patients showed reduced regional gray matter volumes (GMV) in the left caudate nucleus, parts of the anterior cingulate gyrus (ACC) and primary sensorimotor areas (Figure 1). In addition, significant GMV reductions were present in the middle frontal gyrus and the medial frontal gyrus of the right hemisphere. Peak differences in voxel volumes between patients and control subjects and the corresponding Brodmann areas are summarized in Table 1. No regions displayed significant regional increases in GMV for TS patients versus controls.

**Table 1:** Brain areas with regional gray matter volume reductions in TS patients compared to normal subjects

Cluster size	Regions (Brodmann areas)	MNI-Space			*t*-value	*p*-value (SVC)
				
		*x*	*y*	*z*		
254	Left postcentral gyrus (43)	-55	-8	20	5.04	0.009
83	Right precentral gyrus (4)	38	-20	47	4.54	0.031
60	right cingulate gyrus (32)	9	22	31	4.01	0.049
224	Left caudate (body)	-15	14	13	4.00	0.018
	Left caudate (head)	-10	11	3	3.54	
57	Left middle frontal gyrus (6)	-36	12	56	4.00	0.018
180	Left precentral gyrus (4)	-45	-18	36	3.96	0.020
	Left precentral gyrus (4)	-52	-15	40	3.52	
58	Right middle frontal gyrus (6)	44	6	57	3.96	0.020
152	Right precentral gyrus (6)	59	-13	38	3.95	0.020
43	Right precentral gyrus (4)	45	-19	38	3.95	0.017
25	Right postcentral gyrus (3)	41	-26	58	3.68	0.014
14	Right medial frontal gyrus (8)	10	29	48	3.62	0.042
20	Left cingulate gyrus (32)	-9	12	39	3.61	0.043

Cluster size = number of voxels; x, y, z = Coordinates (in mm) of significant local maxima are given for information in MNI space (Montreal Neurological Institute, ). Significant local maxima more than 8 mm apart were translated to brain anatomy by converting their coordinates from MNI space to Talairach space  allowing for a search radius of 9 mm for nearest gray matter. For regions where an effect was hypothesized (see introduction), a small volume correction (SVC) was performed. P values are given after family wise error (FWE) correction for the particular volume. No regions displayed significant regional increases in GMV for TS patients versus controls.

**Figure 2:**
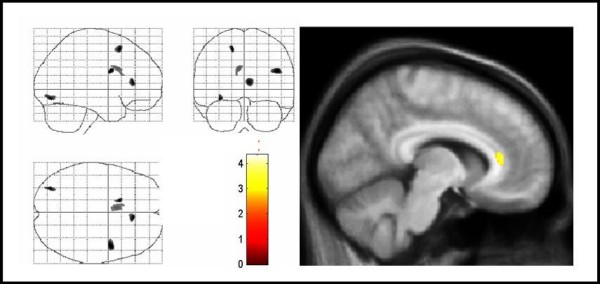
**Areas of decreased regional white matter volumes in TS patients compared with controls**. Areas of decreased regional white matter volumes in TS patients compared with controls. The same conventions apply as for figure 1. Significant voxels were found in the anterior corpus callosum, right inferior frontal gyrus, and left superior frontal gyrus (corrected P < .05). Note that changes in the left lingual gyrus, which were not significant, are also displayed. TS = Tourette syndrome.

**Figure 3:**
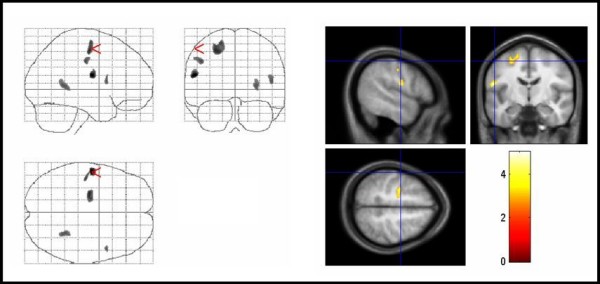
**Areas of increased regional white matter volumes in TS patients compared with controls**. Areas of increased regional white matter volumes in TS patients compared with controls. The same conventions apply as for figure 1. Significant voxels were found in the left primary sensorimotor cortex, and left middle frontal gyrus (corrected P < 0.05). Note that changes in the right parahippocampal gyrus, which were not significant, are also displayed. TS = Tourette syndrome.

For white matter volumes (WMV), there were significant regional decreases in patients compared with controls in the white matter below the right inferior frontal gyrus, the left superior frontal gyrus, and in the anterior corpus callosum (Figure 2, Table 1). Regional WMV increases were found in the left middle frontal gyrus and primary sensorimotor areas on the left side (Figure 3, Table 2).

**Table 2:** Brain areas with regional white matter volume changes and MTR reductions (TS versus controls)

Cluster size	Regions (Brodmann areas)	MNI-Space			*t*-value	*p*-value (SVC)
				
		*x*	*y*	*z*		
**WMV: controls > TS**						
157	Right inferior frontal gyrus (9)	44	3	25	4.35	0.010
81	Left superior frontal gyrus (6)	-16	15	54	4.05	0.021
33	Left precentral gyrus (6)	-25	-20	70	3.93	0.027
115	Right anterior corpus callosum	7	34	10	3.92	0.028
79	Left lingual gyrus (18)	-32	-77	-12	3.82	-
**WMV: TS > controls**						
267	Left postcentral gyrus (43)	-54	-10	20	5.02	0.005
344	Left precentral gyrus (6)	-26	-12	53	4.00	0.023
	Left middle frontal gyrus (6)	-19	-11	62	3.74	0.031
42	Left precentral gyrus (4)	-45	-18	38	3.60	0.056
34	Right parahippocampal gyrus (30)	27	-46	6	3.49	0.060
10	Right precentral gyrus (44)	49	10	11	3.39	0.085
**MTR maps: controls > TS**						
99	Right medial frontal gyrus (9)	17	32	37	3,75	0.036
33	Right inferior frontal gyrus (46)	40	32	12	3,65	0.045
33	Left inferior frontal gyrus (46)	-41	24	13	3,63	0.041
17	Right cingulate gyrus (32)	11	22	43	3,45	0.048

MNI = Montreal Neurological Institute, SVC = small volume correction, WMV = white matter volume, MTR = magnetization transfer ratio, TS = Tourette syndrome. No regions displayed significant regional increases in MTR maps for TS patients versus controls.

#### Magnetization transfer imaging

**Figure 4:**
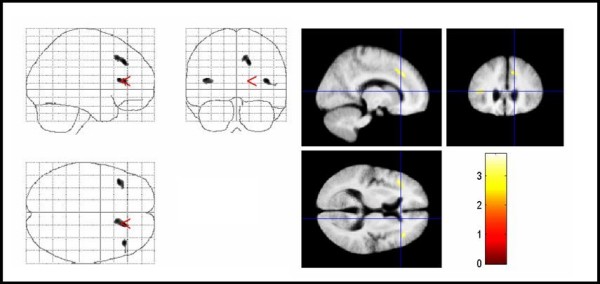
**Areas of decreased MTR in TS patients compared with controls**. Areas of decreased MTR in TS patients compared with controls. The significant results are superimposed on the averaged normalized MTR maps of the study population (thresholded at uncorrected P < .005 for display purpose only). Significant voxels were found in the right medial frontal gyrus, inferior frontal gyrus bilaterally, and the right cingulate gyrus (corrected P < .05). TS = Tourette syndrome, MTR = magnetization transfer ratio.

Patients displayed significant regional MTR map reductions in the right medial frontal gyrus, the inferior frontal gyrus bilaterally and the right ACC compared with controls. No significant increases in MTR maps were found in TS patients compared to controls (Figure 4, Table 2).

### Correlations with clinical scores

#### VBM

**Figure 5:**
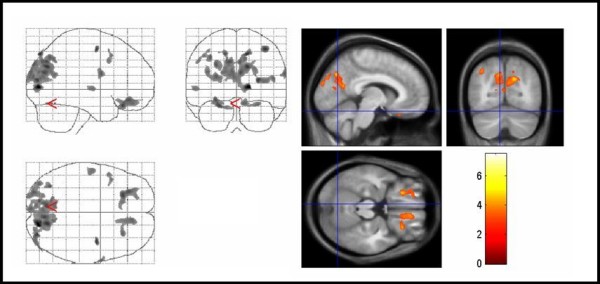
**Areas with negative correlation between regional gray matter volumes and YGTSS score in TS patients**. Areas with negative correlation between regional gray matter volumes and YGTSS score in TS patients. The same conventions apply as for figure 1. Significant voxels for GMV reductions with higher YGTSS scores were found in the orbitfrontal cortex, and the parietal-temporal-occipital association cortex bilaterally (corrected P < .05). YGTSS = Yale global tic severity scale, TS = Tourette syndrome.

Using the SPM2 model 'single subjects, covariates only' the YGTSS score was negatively correlated with GMV, indicating that increased TS symptom severity was associated with regional volume reductions in the orbitofrontal cortex bilaterally. Additionally, volume reductions in parts of the parietal-temporal-occipital association cortex bilaterally were found (Figure 5, Table 3). There was no correlation between WMV and YGTSS.

**Table 3:** Brain areas with negative correlation between regional gray matter volumes and YGTSS scores in TS

Cluster size	Regions (Brodmann areas)	MNI-Space			*t*-value	*p*-value (SVC)
				
		*x*	*y*	*z*		
434	Right cuneus (17)	17	-83	3	7.49	0.001
142	Left insula	-42	-39	17	6.38	0.004
728	Right precuneus (7)	15	-71	30	5.15	0.023
134	Right middle frontal gyrus (6)	38	-5	55	5.03	0.028
171	Right middle occipital gyrus (19)	29	-85	13	4.93	-
431	Left inferior frontal gyrus (11)	-23	30	-21	4.76	0.042
	Left middle frontal gyrus (11)	-28	38	-14	4.50	0.021
	Left superior frontal gyrus (11)	-30	46	-16	3.76	0.030
45	Right middle temporal gyrus (21)	64	-2	-16	4.62	-
33	Right superior frontal gyrus (6)	18	3	70	4.58	0.020
266	Left precuneus (7)	-9	-73	38	4.56	0.021
73	Left anterior cingulate (32)	-10	34	-6	4.51	0.020
101	Left Cuneus (19)	-27	-89	33	4.42	0.040
70	left superior parietal lobule (7)	-35	-68	43	4.26	0.033
70	Right precentral gyrus (6)	64	1	22	4.23	0.035
24	Left superior temporal gyrus (38)	-51	17	-14	4.18	-
21	Left medial frontal gyrus (10)	-11	53	18	4.16	0.039
41	Left precentral gyrus (6)	-42	-4	47	4.03	0.047
40	Right middle frontal gyrus (11)	27	29	-20	4.01	0.048
105	Right inferior frontal gyrus (11)	9	42	-18	4.00	0.050
41	Left superior occipital gyrus (19)	-38	-82	24	3.99	-

MNI = Montreal Neurological Institute, SVC = small volume correction.

#### Magnetization transfer imaging

**Figure 6:**
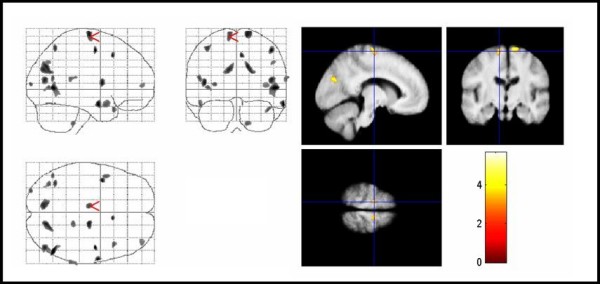
**Areas with negative correlation between MTR maps and YGTSS score in TS patients**. Areas with negative correlation between MTR maps and YGTSS score in TS patients. The same conventions apply as for figure 4. Significant voxels for MTR map reductions with higher YGTSS scores were found in right anterior cingulate gyrus, parietal-temporal-occipital association cortex, and superior frontal gyrus bilaterally (corrected P < .05). Note that changes in temporal areas, which were not significant according to our hypothesis, are also displayed. MTR = magnetization transfer ratio, YGTSS = Yale global tic severity scale, TS = Tourette syndrome.

YGTSS scores correlated negatively with MTR maps in the superior frontal gyrus bilaterally and the left inferior frontal lobe. In addition, negative correlations were found in the right ACC and parts of the parietal-temporal-occipital association cortex (Figure 6, Table 4).

**Table 4:** Brain areas with negative correlation between MTR maps and YGTSS scores in TS patients

Cluster size	Regions (Brodmann areas)	MNI-Space			*t*-value	*p*-value (SVC)
				
		*x*	*y*	*z*		
262	Right middle temporal gyrus (37)	51	-63	2	5.67	-
83	Right precentral gyrus (6)	47	-7	53	5.57	0.006
103	Right inferior temporal gyrus (21)	63	-3	-19	5.45	-
121	Left precentral gyrus (44)	-42	3	10	4.97	0.015
253	Right superior frontal gyrus (6)	14	-15	74	4.90	0.017
42	Right cingulate gyrus (32)	7	16	46	4.86	0.018
235	Right cuneus (7)	15	-73	31	4.82	0.019
	Right precuneus (31)	20	-69	25	4.30	0.019
153	Right middle temporal gyrus (39)	47	-74	13	4.65	0.024
220	Left precuneus (31)	-10	-76	27	4.49	0.031
48	Left superior parietal lobule (7)	-39	-61	56	4.25	0.045
14	Right inferior parietal lobule (40)	42	-49	61	4.06	0.060
18	Left middle occipital gyrus (18)	-29	-93	8	3.99	-
81	Left superior frontal gyrus (6)	-9	-16	74	3.98	0.068
17	Left middle temporal gyrus (19)	-50	-64	15	3.91	-
21	Right superior temporal gyrus (38)	42	18	-27	3.88	-
14	Left middle temporal gyrus (21)	-49	6	-20	3.81	-

MNI = Montreal Neurological Institute, SVC = small volume correction.

## Discussion

Recent structural studies using conventional volumetric MRI yielded conflicting results regarding TS-related regional brain volume alterations. To avoid influences from age, gender, co-morbidities, and medication, we investigated a carefully selected group of TS patients by means of VBM and – for the first time – by MTI analyzed on a voxel-by-voxel basis. Tissue alterations in comparison to controls were detected in the supplementary motor area (SMA), the premotor cortex, the sensorimotor cortex, the prefrontal cortex, the left caudate nucleus, and the ACC. Tic severity was negatively correlated with tissue alterations in the orbitofrontal cortex, parts of the parietal-temporal-occipital association cortex bilaterally, the SMA, and the right ACC.

Current concepts of basal ganglia loops suggest different parallel circuits that connect frontal association areas with basal ganglia. It is thought that these circuits are involved in the selection, programming, initiation, and control of movement [29]. The hypothesis of an involvement of these loops in TS [30] is further corroborated by our results as we detected changes in several parts of these circuits. In contrast to other volumetric studies, but in agreement with data obtained from functional investigations [16,31,32], we detected the most prominent changes not in the basal ganglia but in the prefrontal cortex and the ACC. Our data, therefore, are in line with Peterson's measurements [2] in adult patients demonstrating decreased frontal brain volumes. However, these results are in contrast to a recent VBM study [14] in adult TS patients that demonstrated increased regional GMV in the left midbrain and failed to detect alterations in other cortical areas. In this study, however, white matter volumes were not assessed. These discrepancies might be related to differences in the cohorts of patients and methodological factors within the data analysis process [26]. Data from all other aforementioned volumetric MRI studies – although several of them as well demonstrated frontal lobe abnormalities – cannot be directly compared to our results as most data were obtained from children.

Miscellaneous studies are in line with our findings demonstrating most significant changes in frontal areas [16,31-33]. Therefore, it has been suggested that TS is associated with abnormal pattern-related increases in metabolic activity in different frontal regions that are involved in the execution of movement (e.g., sensorimotor and lateral premotor cortex, SMA) [32]. Thus, anomalous frontal lobe associational and projection fiber bundles may be the basis for basal ganglia abnormalities in TS [12]. This assumption, in turn, is in agreement with the finding that the dorsolateral prefrontal cortex projects primarily to the dorsolateral head of the caudate nucleus [3].

Only a limited number of volumetric MRI studies is available investigating limbic regions in TS. Disturbances in the anterior cingulate (limbic) cortex, however, are likely to play a critical role in TS pathology [34,35]. Firstly, stimulation of the ACC is associated with the generation of involuntary vocalizations. Secondly, cingulate epilepsy is combined with complex motor automatisms. Thirdly, the ACC has numerous interconnections with the prefrontal and orbitofrontal cortex, motor systems, other limbic regions, and the striatum – regions that are suggested to be involved in TS pathology. Fourthly, the ACC has an important role not only in movement, but also in the initiation and motivation of goal-directed-behaviour. Fifthly, activation or dysfunction of the ACC can be associated with aberrant social behaviour and psychopathic behaviours such as OCD [36,37]. From functional neuroimaging studies it is suggested that tic generation is caused not only by basal ganglia and frontal cortex dysregulation but also by alterations in the cingulate gyrus [16,32,34]. While an increased ACC activity has been found during tic suppression [31], an ACC hypoperfusion was detected at rest without tic suppression [34]. A positive correlation between tic frequency and ACC activity has been demonstrated [30]. Using VBM, GMV reductions in the left hippocampal gyrus have been detected in a boys-only group [15]. Our results of reduced gray and white matter cingulate volumes and a negative correlation between tic severity and tissue alterations in the right ACC are in agreement with these data.

Since the ACC seems to be physiologically under control of the prefrontal cortex [38], it can be speculated that in TS, abnormalities in frontal lobe fiber bundles result in a desinhibition of the ACC. A subsequent autonomous discharge of the ACC might cause motor and vocal tics, OCD, and other behavioural problems. Tics are often stimulated by simply thinking about tics or by surrounding factors. These specific characteristics of tics might be well explained by a dysfunctional ACC, since it is thought that the integration of thought, motivation, and emotion with movement is an important cingulate function [36].

Regarding basal ganglia volumes our results are in agreement with data from Peterson's large study demonstrating reduced caudate nuclei volumes in adults, although we detected changes only on the left side [2]. Comparable to our findings, only left-sided caudate abnormalities have been reported in several positron emission tomography (PET) and single photon emission computed tomography (SPECT) studies [32,34,39]. It can be assumed that basal ganglia volumes are increased in children but decreased in adults [2]. However, it remains unclear whether caudate volumes decrease with age or whether reduced volumes represent a compensatory response to the presence of tics. Since the striatum is involved not only in the initiation and execution of movements but also in behavioural functions [34] it is conceivable that the complex clinical manifestation of TS – at least in part – is caused by a dysfunctional caudate nucleus.

Our finding of reduced WMV in the corpus callosum is in agreement with one study in adult TS patients [40] but contrasts to other studies [3,41]. In adults an increased corpus callosum size may be associated with co-morbidities [41].

In addition, we found a significantly lower amount of regional GMV in the parietal lobe on the left side. In both, VBM and MTI, YGTSS correlated negatively with parts of the parietal-temporal-occipital association cortex bilaterally. Comparable results has been described before using functional MRI (fMRI) [16], PET [32], and SPECT [34]. Reduced metabolic activation and hypoperfusion in the medial and lateral temporal region were found to be related to the severity of tics [30,32,34]. Furthermore, functional abnormalities in the superior and inferior parietal lobules and the parietal operculum have been described before and at tic onset, respectively [16,30,32,42]. In the parietal-temporal-occipital association cortex higher perceptual functions relate to somatic sensations, hearing and vision to form complex perceptions from these different sensory modalities. The superior parietal lobule functionally connects to the SMA and the prefrontal cortex and is thought to perform complex integrative functions related to the organization and initiation of movement [42]. Therefore, abnormal connections between the superior parietal lobule, premotor and limbic regions may underlie premonitory sensory urges preceding tics in TS [42].

Recently, an increase of the regional fractional anisotropy (an indicator of microstructural integrity of brain tissue) has been shown in the white matter underlying the left somatosensory cortex in adult TS patients compared to controls [43]. Additionally, this region showed an inverse linear relationship with tic severity. This is well in line with our finding of increased WMV in the sensorimotor cortex, and is in good agreement with the concept of an involvement of somatosensory pathways in TS. Apart from a recent structural MRI study which demonstrated thinning of the sensorimotor cortex in children with TS [44], behavioural and neurophysiological studies provide further evidence for abnormal sensory-motor processing in TS patients [45,46]. These structural alterations may represent an adaptive response of the somatosensory system to abnormal input from fronto-striatal circuits.

Some limitations of this study have to be addressed. One might argue that in TS patients tic severity typically fluctuates over time whereas the tic score on a scale such as the YGTSS relates to symptoms present over the last weeks prior assessment. However, for different reasons we believe that our tic rating indeed reflects disease severity and, therefore, is suitable for a correlation in an MRI morphometric study: (1) the mean age of our sample was quite high (mean age = 30.4) and it is well known that in adults at that age tics do not fluctuate as much as in children and adolescents, (2) since in our sample mean tic severity was quite moderate (28.8 according to YGTSS), patients were able to remain either drug-naïve or drug-free for a longer period of time. This fact further supports the assumption that the patients' tics did not change markedly during the past.

From a methodological point of view, there are potential problems in the use of voxel-by-voxel analysis. First, this analysis was originally intended for use in large samples and requires smoothing of the images, with loss of resolution for small structures. Furthermore, the large number of comparisons requires corrections that greatly reduce the power of the study. This could explain why we did not find significant VBM and MTR differences between TS and controls on a whole brain analysis. Secondly, the small size of our group makes the results vulnerable to type I or type II errors, although in recent VBM and MTI studies [18,26] a similar number of patients was suitable to detect regional differences compared to normal controls. Thirdly, the location of the MTI and VBM abnormalities overlapped in some regions but was not identical. This apparent inconsistency could be due to the use of a volume of interest (SVC) that, while increasing the power of the analysis by reducing the number of comparisons, may exclude potentially abnormal areas. Furthermore, the segmented VBM images, with a slice thickness of 1 mm, have higher resolution and ability to differentiate white and gray matter abnormalities than the MTI images with a slice thickness of 3 mm. In addition, segmented gray and white matter images have a smaller search volume, which affects sensitivity.

The MTR is largely dependent on the macromolecular density of cell membranes and phospholipids, and gray matter MTR decreases in our TS patients are likely to reflect decreases in the size and number of neurons and dendritic density, while those in the white matter are likely to reflect myelin changes and/or reduced axonal density [47]. The neuropathological counterparts of our VBM findings are less clear. Potential correlates include a simple change in cell size, growth or atrophy of neurons or glia, as well as changes in the intra-cortical axonal architecture (synaptogenesis). Previously, it has been suggested that disturbances in the maturation of fronto-striatal systems contribute to the development, persistence, and severity of tics in adult TS patients [48]. This assumption is supported by the fact that MTR decreases in the prefrontal cortex, which maturates later than other regions [49], could be indicative for alterations in the degree of myelinization. Furthermore, one might argue that results obtained from adult patients, as in the present study, reflect adaptive mechanisms to compensate for impairments in other brain regions. In line with this, recent studies also suggested that some pathological abnormalities as seen in VBM studies could be due to adaptive neuronal plasticity [50]. However, the problem of separating cause and effect in cross-sectional studies is presently unsolvable.

## Conclusion

This is the first study using MTI to investigate structural brain abnormalities in TS patients. In addition, we used VBM to investigate regional gray and white matter volume changes in a carefully selected group of adult, unmedicated male TS patients without co-morbidities and age- and sex-matched normal controls. Our findings support the hypothesis that alterations in fronto-striatal circuitries underlie TS pathology – even in patients without co-morbidities. We suggest that TS is primarily caused by a dysfunction in prefrontal cortex areas rather than the basal ganglia. It can be speculated that anomalous frontal lobe association and projection fiber bundles disinhibit the ACC and are the basis for basal ganglia dysfunction in TS.

## Authors' contributions

KMV and TP drafted the manuscript. JK and TP performed the data acquisition and pre- processing of the data. KMV designed, analyzed, and collected the clinical data set. TP and JK designed the MRI study and performed the statistical analysis. JG participated in the statistical analysis of the data. KMV, RD and HMH participated in the design and coordination of the study. RD and HMH were involved in the interpretation of results and general conclusions. All authors read and approved the final manuscript.

## References

[B1] Peterson BS, Riddle MA, Cohen DJ, Katz LD, Smith JC, Leckman JF (1993). Human basal ganglia volume asymmetries on magnetic resonance images. Magn Reson Imaging.

[B2] Peterson BS, Thomas P, Kane MJ, Scahill L, Zhang H, Bronen R (2003). Basal Ganglia volumes in patients with Gilles de la Tourette syndrome. Arch Gen Psychiatry.

[B3] Moriarty J, Varma AR, Stevens J, Fish M, Trimble MR, Robertson MM (1997). A volumetric MRI study of Gilles de la Tourette's syndrome. Neurology.

[B4] Singer HS, Reiss AL, Brown JE, Aylward EH, Shih B, Chee E (1993). Volumetric MRI changes in basal ganglia of children with Tourette's syndrome. Neurology.

[B5] Zimmerman AM, Abrams MT, Giuliano JD, Denckla MB, Singer HS (2000). Subcortical volumes in girls with tourette syndrome: support for a gender effect. Neurology.

[B6] Hyde TM, Stacey ME, Coppola R, Handel SF, Rickler KC, Weinberger DR (1995). Cerebral morphometric abnormalities in Tourette's syndrome: a quantitative MRI study of monozygotic twins. Neurology.

[B7] Bloch MH, Leckman JF, Zhu H, Peterson BS (2005). Caudate volumes in childhood predict symptom severity in adults with Tourette syndrome. Neurology.

[B8] Castellanos FX, Giedd JN, Hamburger SD, Marsh WL, Rapoport JL (1996). Brain morphometry in Tourette's syndrome: the influence of comorbid attention-deficit/hyperactivity disorder. Neurology.

[B9] Peterson BS, Leckman JF, Tucker D, Scahill L, Staib L, Zhang H (2000). Preliminary findings of antistreptococcal antibody titers and basal ganglia volumes in tic, obsessive-compulsive, and attention deficit/hyperactivity disorders. Arch Gen Psychiatry.

[B10] Giedd JN, Rapoport JL, Garvey MA, Perlmutter S, Swedo SE (2000). MRI assessment of children with obsessive-compulsive disorder or tics associated with streptococcal infection. Am J Psychiatry.

[B11] Peterson BS, Staib L, Scahill L, Zhang H, Anderson C, Leckman JF (2001). Regional brain and ventricular volumes in Tourette syndrome. Arch Gen Psychiatry.

[B12] Fredericksen KA, Cutting LE, Kates WR, Mostofsky SH, Singer HS, Cooper KL (2002). Disproportionate increases of white matter in right frontal lobe in Tourette syndrome. Neurology.

[B13] Kates WR, Frederikse M, Mostofsky SH, Folley BS, Cooper K, Mazur-Hopkins P (2002). MRI parcellation of the frontal lobe in boys with attention deficit hyperactivity disorder or Tourette syndrome. Psychiatry Res.

[B14] Garraux G, Goldfine A, Bohlhalter S, Lerner A, Hanakawa T, Hallett M (2006). Increased midbrain gray matter in Tourette's syndrome. Ann Neurol.

[B15] Kassubek J, Juengling FD, Ludolph AG (2006). Heterogeneity of voxel-based morphometry findings in Tourette's syndrome: an effect of age?. Ann Neurol.

[B16] Bohlhalter S, Goldfine A, Matteson S, Garraux G, Hanakawa T, Kansaku K (2006). Neural correlates of tic generation in Tourette syndrome: an event-related functional MRI study. Brain.

[B17] Pendlebury ST, Lee MA, Blamire AM, Styles P, Matthews PM (2000). Correlating magnetic resonance imaging markers of axonal injury and demyelination in motor impairment secondary to stroke and multiple sclerosis. Magn Reson Imaging.

[B18] Eckert T, Sailer M, Kaufmann J, Schrader C, Peschel T, Bodammer N (2004). Differentiation of idiopathic Parkinson's disease, multiple system atrophy, progressive supranuclear palsy, and healthy controls using magnetization transfer imaging. Neuroimage.

[B19] Cercignani M, Bozzali M, Iannucci G, Comi G, Filippi M (2001). Magnetisation transfer ratio and mean diffusivity of normal appearing white and grey matter from patients with multiple sclerosis. J Neurol Neurosurg Psychiatry.

[B20] Lerner A, Bagic A, Boudreau EA, Hanakawa T, Pagan F, Mari Z (2007). Neuroimaging of neuronal circuits involved in tic generation in patients with Tourette syndrome. Neurology.

[B21] Leckman JF, Riddle MA, Hardin MT, Ort SI, Swartz KL, Stevenson J (1989). The Yale Global Tic Severity Scale: initial testing of a clinician-rated scale of tic severity. J Am Acad Child Adolesc Psychiatry.

[B22] Goodman WK, Price LH, Rasmussen SA, Mazure C, Fleischmann RL, Hill CL (1989). The Yale-Brown Obsessive Compulsive Scale. I. Development, use, and reliability. Arch Gen Psychiatry.

[B23] Retz-Junginger P, Retz W, Blocher D, Stieglitz RD, Georg T, Supprian T (2003). [Reliability and validity of the Wender-Utah-Rating-Scale short form. Retrospective assessment of symptoms for attention deficit/hyperactivity disorder]. Nervenarzt.

[B24] Woods RP, Mazziotta JC, Cherry SR (1993). MRI-PET registration with automated algorithm. J Comput Assist Tomogr.

[B25] Schiffer B, Peschel T, Paul T, Gizewski E, Forsting M, Leygraf N (2007). Structural brain abnormalities in the frontostriatal system and cerebellum in pedophilia. J Psychiatr Res.

[B26] Grosskreutz J, Kaufmann J, Fradrich J, Dengler R, Heinze HJ, Peschel T (2006). Widespread sensorimotor and frontal cortical atrophy in Amyotrophic Lateral Sclerosis. BMC Neurol.

[B27] Camara E, Bodammer N, Rodriguez-Fornells A, Tempelmann C (2007). Age-related water diffusion changes in human brain: a voxel-based approach. Neuroimage.

[B28] Schiffer B, Peschel T, Paul T, Gizewski E, Forsting M, Leygraf N (2007). Structural brain abnormalities in the frontostriatal system and cerebellum in pedophilia. J Psychiatr Res.

[B29] Alexander GE, Crutcher MD, DeLong MR (1990). Basal ganglia-thalamocortical circuits: parallel substrates for motor, oculomotor, "prefrontal" and "limbic" functions. Prog Brain Res.

[B30] Stern E, Silbersweig DA, Chee KY, Holmes A, Robertson MM, Trimble M (2000). A functional neuroanatomy of tics in Tourette syndrome. Arch Gen Psychiatry.

[B31] Peterson BS, Skudlarski P, Anderson AW, Zhang H, Gatenby JC, Lacadie CM (1998). A functional magnetic resonance imaging study of tic suppression in Tourette syndrome. Arch Gen Psychiatry.

[B32] Eidelberg D, Moeller JR, Antonini A, Kazumata K, Dhawan V, Budman C (1997). The metabolic anatomy of Tourette's syndrome. Neurology.

[B33] Minzer K, Lee O, Hong JJ, Singer HS (2004). Increased prefrontal D2 protein in Tourette syndrome: a postmortem analysis of frontal cortex and striatum. J Neurol Sci.

[B34] Moriarty J, Costa DC, Schmitz B, Trimble MR, Ell PJ, Robertson MM (1995). Brain perfusion abnormalities in Gilles de la Tourette's syndrome. Br J Psychiatry.

[B35] Weeks RA, Turjanski N, Brooks DJ (1996). Tourette's syndrome: a disorder of cingulate and orbitofrontal function?. QJM.

[B36] Devinsky O, Morrell MJ, Vogt BA (1995). Contributions of anterior cingulate cortex to behaviour. Brain.

[B37] Singer HS, Dela Cruz PS, Abrams MT, Bean SC, Reiss AL (1997). A Tourette-like syndrome following cardiopulmonary bypass and hypothermia: MRI volumetric measurements. Mov Disord.

[B38] Cohen RM, Semple WE, Gross M, King AC, Nordahl TE (1992). Metabolic brain pattern of sustained auditory discrimination. Exp Brain Res.

[B39] Braun AR, Stoetter B, Randolph C, Hsiao JK, Vladar K, Gernert J (1993). The functional neuroanatomy of Tourette's syndrome: an FDG-PET study. I. Regional changes in cerebral glucose metabolism differentiating patients and controls. Neuropsychopharmacology.

[B40] Peterson BS, Leckman JF, Duncan JS, Wetzles R, Riddle MA, Hardin MT (1994). Corpus callosum morphology from magnetic resonance images in Tourette's syndrome. Psychiatry Res.

[B41] Plessen KJ, Gruner R, Lundervold A, Hirsch JG, Xu D, Bansal R (2006). Reduced white matter connectivity in the corpus callosum of children with Tourette syndrome. J Child Psychol Psychiatry.

[B42] Jeffries KJ, Schooler C, Schoenbach C, Herscovitch P, Chase TN, Braun AR (2002). The functional neuroanatomy of Tourette's syndrome: an FDG PET study III: functional coupling of regional cerebral metabolic rates. Neuropsychopharmacology.

[B43] Thomalla G, Siebner HR, Jonas M, Baumer T, Biermann-Ruben K, Hummel F (2009). Structural changes in the somatosensory system correlate with tic severity in Gilles de la Tourette syndrome. Brain.

[B44] Sowell ER, Kan E, Yoshii J, Thompson PM, Bansal R, Xu D (2008). Thinning of sensorimotor cortices in children with Tourette syndrome. Nat Neurosci.

[B45] Nowak DA, Rothwell J, Topka H, Robertson MM, Orth M (2005). Grip force behavior in Gilles de la Tourette syndrome. Mov Disord.

[B46] Lemay M, Termoz N, Lesperance P, Chouinard S, Rouleau GA, Richer F (2007). Postural control anomalies in children with Tourette syndrome. Exp Brain Res.

[B47] Bruno SD, Barker GJ, Cercignani M, Symms M, Ron MA (2004). A study of bipolar disorder using magnetization transfer imaging and voxel-based morphometry. Brain.

[B48] Marsh R, Zhu H, Wang Z, Skudlarski P, Peterson BS (2007). A developmental fMRI study of self-regulatory control in Tourette's syndrome. Am J Psychiatry.

[B49] Yurgelun-Todd D (2007). Emotional and cognitive changes during adolescence. Curr Opin Neurobiol.

[B50] May A, Gaser C (2006). Magnetic resonance-based morphometry: a window into structural plasticity of the brain. Curr Opin Neurol.

